# Two new genera of metalmark butterflies of North and Central America (Lepidoptera, Riodinidae)

**DOI:** 10.3897/zookeys.729.20179

**Published:** 2018-01-16

**Authors:** Marysol Trujano-Ortega, Uri Omar García-Vázquez, Curtis J. Callaghan, Omar Ávalos-Hernández, Moisés Armando Luis-Martínez, Jorge Enrique Llorente-Bousquets

**Affiliations:** 1 Museo de Zoología, Departamento de Biología Evolutiva, Facultad de Ciencias, Universidad Nacional Autónoma de México, Apdo. Postal 70–399, México 04510, Ciudad de México, México; 2 Posgrado en Ciencias Biológicas, Universidad Nacional Autónoma de México, México; 3 Facultad de Estudios Superiores Zaragoza, Universidad Nacional Autónoma de México, Batalla 5 de Mayo s/n, Ejército de Oriente, Ciudad de México 09230, México; 4 Casa Picapau, Floresta de la Sabana. Carrera 7, 237–04, Bogotá, Colombia

**Keywords:** *Apodemia*, molecular phylogeny, Papilionoidea, semiarid regions, taxonomy

## Abstract

Two new genera of Riodinidae (Insecta: Lepidoptera) are described, *Neoapodemia* Trujano-Ortega, **gen. n.** (*Neoapodemia nais* (W. H. Edwards, 1876), **comb. n.**, *N. chisosensis* Freeman, 1964, **comb. n.**) and *Plesioarida* Trujano-Ortega & García-Vázquez, **gen. n.** (*Plesioarida palmerii palmerii* (W. H. Edwards, 1870), **comb. n.**, *P. palmerii arizona* (Austin, [1989]), **comb. n.**, *P. palmerii australis* (Austin, [1989]), **comb. n.**, *P. hepburni hepburni* (Godman & Salvin, 1886), **comb. n.**, *P. hepburni remota* (Austin, 1991), **comb. n.**, *P. murphyi* (Austin, [1989]), **comb. n.**, *P. hypoglauca hypoglauca* (Godman & Salvin, 1878), **comb. n.**, *P. hypoglauca wellingi* (Ferris, 1985), **comb. n.**, *P. walkeri* (Godman & Salvin, 1886), **comb. n.**, *P. selvatica* (De la Maza & De la Maza, 2017), **comb. n.**). *Neoapodemia* Trujano-Ortega, **gen. n.** is distributed in the southwestern USA and northeastern Mexico, while *Plesioarida* Trujano-Ortega & García-Vázquez, **gen. n.** is present from the southern USA to Central America. Species of these genera were previously classified as *Apodemia* C. Felder & R. Felder but molecular and morphological evidence separate them as new taxa. Morphological diagnoses and descriptions are provided for both new genera, including the main distinctive characters from labial palpi, prothoracic legs, wing venation and genitalia, as well as life history traits. A molecular phylogeny of one mitochondrial gene (COI) and two nuclear genes (EF-1a and wg) are also presented of most species of *Apodemia*, *Neoapodemia* Trujano-Ortega, **gen. n.**, *Plesioarida* Trujano-Ortega & García-Vázquez, **gen. n.**, and sequences of specimens from all tribes of Riodinidae. We compare the characters of *Apodemia*, *Neoapodemia* Trujano-Ortega, **gen. n.** and *Plesioarida* Trujano-Ortega & García-Vázquez, **gen. n.** and discuss the differences that support the description of these new taxa. This is a contribution to the taxonomy of the Riodinidae of North America of which the generic diversity is greater than previously recognized.

## Introduction

Butterflies of the family Riodinidae exhibit a great variation in wing shape, color, and pattern. They represent more than 8% of all butterflies and are found mainly in the New World, where they comprise approximately 133 genera and more than 1350 described species arranged in two subfamilies, Riodininae (1200 species) and Euselasiinae (176 species) (Espeland et al. 2015). This considerable diversity has caused some taxonomic confusion. While the position of the family Riodinidae in the phylogeny of Lepidoptera is well-resolved (Saunders 2010), the relationships between genera within the family are poorly understood (Kristensen 1976, de Jong et al. 1996, Ackery et al. 1999, Wahlberg et al. 2005). In recent years, this family has been subject to several studies attempting to clarify its internal relationships and taxonomy (Hall and Harvey 2002, Hall 2003, Sperling 2003, Campbell and Pierce 2003, Saunders 2010, Espeland et al. 2015).

*Apodemia* C. Felder & R. Felder, [1865] is a genus living in arid and semiarid regions of western North America ranging from southern Canada and the northeastern USA to Central America, with only one South American species in Brazil. DeVries (1997) noted that the genus is increasingly rare towards the southern end of its distribution. This genus currently contains 36 taxa with 16 described species and 26 subspecies, most of which belong to the *A. mormo* complex (DeVries 1997, Pelham 2008, Proshek 2011, Warren et al. 2017). Thirteen (81%) of the 16 species of *Apodemia* are present in Mexico, of which seven species and four subspecies are endemic to the country (Callaghan and Lamas 2004, Llorente-Bousquets et al. 2013, De la Maza and De la Maza 2017a, b, Warren et al. 2017).

The original description of *Apodemia* is brief and refers to the antennal characters (Felder and Felder 1864–1867) and wing patterns. For this reason, following the original description, other authors defined additional characters to distinguish the genus (Godman and Salvin 1878–1901, DeVries 1997) and its type species *A. mormo mormo* C. Felder & R. Felder (Opler and Powell 1961). These characters include a less atrophied venation in the anterior wings, joint point of the trochanter and the coxa, the presence of two rows of spines in the tarsi, the large palpi and the sagittate signa in female genitalia. DeVries (1997) mentions that although this genus includes some butterflies similar to those of *Lasaia* H. Bates and *Emesis* [Fabricius], it can be distinguished by the leg configurations and the distinctive features of the veins.

Most of the taxonomic studies of *Apodemia* were published during the 20^th^ century, and more than half of these are descriptions of subspecies (Emmel and Emmel 1998, Emmel et al. 1998). Recently, the genetic structure of some populations of *A. mormo* and its subspecies in the USA and Canada were studied (Proshek 2011, Proshek et al. 2013, 2015).

The first data on the phylogenetic relations between the species of *Apodemia* is presented based on molecular data. Also, two new genera are described that emerged from the phylogenetic analysis and are recognizable by their morphology.

## Materials and methods

### Taxon sampling

Eighty male specimens of 15 of the 16 species in the genus *Apodemia* and five *Emesis* species were examined. The material was collected in 2015–2017 in Mexico and gathered from Mexican as well as international scientific collections (Suppl. material 1). These specimens were selected in order to cover the phylogenetic diversity and the geographic distribution of the genus, with an emphasis on type localities. Specimens from *A. selvatica* De la Maza & De la Maza were not reviewed; however, its characteristics are discussed based on the original description (De la Maza and De la Maza, 2017b). Specimens examined came from the following scientific collections: Colección Nacional de Insectos del Instituto de Biología, México (CNIN-IBUNAM), Colección Lepidopterológica del Museo de Zoología de la Facultad de Ciencias, UNAM, Mexico (MZFC); Colección Lepidopterológica del Museo de Zoología de la FES Zaragoza, UNAM, Mexico (MZFZ); McGuire Center for Lepidoptera and Biodiversity, Florida Museum of Natural History, University of Florida, USA (MGCL) and Colección de Curtis Callaghan, Colombia (CJC).

### Morphological procedures

The length of the right anterior wing from the base to the upper apex was measured. Labial palpi and legs were dissected using an Olympus SZX9 stereoscopic microscope, with a planar objective 1.5. The structures and wings were diaphanized by soaking them in alcohol and 5.25% NaClO solution (bleach). Then they were digitized and sketched using an Olympus DP12 camera attached to the microscope. Morphological terminology follows Comstock and Needham (1918), Harvey and Clench (1980), DeVries (1997), Penz and DeVries (1999, 2006) and Hall (1999, 2005). The following abbreviations were used: forewing (FW), hind wing (HW), dorsal (D), ventral (V). Male genitalia were extracted using an enzymatic digestion technique modified from Knölke et al. (2005). If needed, genitalia were soaked a few seconds in a hot potassium hydroxide solution (KOH 10%) to complete the cleaning. All structures were preserved in micro vials with glycerin solution and acetic acid at 4%. Terminology of genitalia descriptions follows Klots (1956), Eliot (1973), Harvey (1987), Penz and DeVries (1999, 2006) and Hall (2008). Digital images of dorsal and ventral views of the genitalia were taken using focus stacking of light microscopy with a Leica Z16 APO-A stereoscopic microscope, a Leica DFC490 HD camera, and the Leica Application Suite program. Cornuti images were taken with a ZEISS microscope AXIO Zoom. V16, with an AxioCam MRc5 camera, and the Zeiss Efficient Navigation program. All images were taken at the Instituto de Biología-UNAM.

### Molecular procedures

Twenty-six specimens of *Apodemia* including eleven of the 16 currently recognized species were collected. Species not included were *Apodemia chisosensis* H. Freeman, *A. virgulti* (Behr), *A. castanea* (Prittwitz), *A. planeca* De la Maza & De la Maza, and *A. selvatica*. Some species (*Apodemia chisosensis*, *A. castanea*, and *A. virgulti*) were excluded due to the lack of sequences from nuclear genes which are more informative in generic level. Two other species (*A. planeca* and *A. selvatica*) have been recently described (De la Maza and De la Maza 2017a, b) and we did not have fresh tissue suitable for DNA extraction (Suppl. material 2). To evaluate the monophyly of *Apodemia*, sequences from 31 other riodinid species were included, representing all tribes of the family according to the most recent phylogeny of Riodinidae (Espeland et al. 2015). With the exception of three sequences of *Emesis*, other sequences of outgroups were taken from GenBank (Suppl. material 2).

DNA was extracted from legs or abdomen using the DNeasy Blood & Tissue kit (Qiagen, Valencia, CA, USA). Partial sequences of 623 bp of the mitochondrial gene Cytochrome Oxidase I (COI) were obtained. Also, two nuclear loci, 495 bp of the gene Elongation factor 1 α (EF-1a), and 402 bp of wingless (wg) were sequenced. These loci were selected because it has been shown previously they are informative at different levels of divergence within riodinid butterflies (Campbell et al. 2000, Campbell and Pierce 2003, Espeland et al. 2015, Proshek et al. 2015). Primer sequences for COI were taken from Proshek et al. (2013), for EF-1a from Monteiro and Pierce (2001), and for wg from Brower and De Salle (1998). All gene regions were amplified via polymerase chain reaction (PCR) in a 25 µL reaction volume containing 0.5–1.0 µL deoxynucleoside triphosphates (dNTPs; 10 mM), 18–19.25 µL double-distilled water, 0.2–0.5 µL each primer (10 mM), 2.5 µL 1X PCR buffer, 1.2 mM MgCl_2_ (Fisherbrand, Pittsburgh, PA, USA), 0.15 µL Taq DNA polymerase (Fisherbrand), and 1.0–1.5 µL template DNA. For COI, DNA was denatured at 94 °C for 2 min, followed by 38–40 cycles of 94 °C for 30 s, 48–50 °C for 45 s, and 72 °C for 45 s. A final extension phase of 72 °C for 7 min terminated the protocol. For EF-1a and wg, DNA was processed according to a touchdown protocol suggest by Espeland et al. (2015) with an initial denaturation for 3 min at 94 °C, 20 cycles of 94 °C for 50 s, annealing temperature starting at 49 °C and ramping down 0.5° for every cycle for 40 s, 72 °C for 1 min, another 20 cycles of 94 °C for 50 s, annealing temperature (48–52 °C) for 40 s, and 72 °C for 1 min, and a final extension of 72 °C for 5 min. Double-stranded PCR products were checked by electrophoresis on a 1% agarose gel. PCR products were purified with polyethylene glycol precipitation (Lis 1980). DNA templates were sequenced in both directions with the Big Dye Terminator version 3.1 cycle sequencing kit (Applied Biosystems, Inc.) and an ABI 3100 automated DNA sequencer (Applied Biosystems, Inc.) using the amplification primers. Sequences were assembled and edited in the STADEN PACKAGE version 1.6.0 (Whitwham and Bonfield 2005).

### Phylogenetic inferences

Sequences were aligned using the MUSCLE algorithm (Edgar 2004) included in the software MEGA version 7 (Kumar et al. 2016). Sequences were uploaded to GenBank and accession numbers are listed in Suppl. material 2. Prior to the concatenated analysis, independent ML analyses were conducted for each gene. The phylogeny of the concatenated data set (n = 56 individuals, including outgroups) was inferred using Bayesian inference and maximum likelihood (ML) phylogenetic methods. For both methods, partitioned analyses were used to improve phylogenetic accuracy. The best-fitting substitution models and partitioning schemes were selected simultaneously using the Bayesian Information Criterion in the software PARTITIONFINDER version 1.1.1 (Lanfear et al. 2012). Bayesian inference analyses were conducted using MRBAYES version 3.2.1 (Ronquist et al. 2012). Four runs were conducted using the ‘nruns = 4’ command, each with three heated and one cold Markov chains with sampling every 1000 generations for 50 million generations. Output parameters were visualized using TRACER version 1.4 (Rambaut and Drummond 2007) to identify stationarity and convergence. Convergence between runs was assessed using AWTY (Nylander et al. 2008). After discarding the first 12.5 million generations (25%) as burn-in, parameter values of the samples were summarized from the posterior distribution on the maximum clade credibility tree using TREEANNOTATOR version 1.4.8 (Drummond and Rambaut 2007) with the posterior probability limit set to 0.1 and mean node heights summarized. Maximum likelihood analyses were conducted using RAxML version 7.2.6 (Stamatakis 2006) under the GTRCAT model, with 1000 nonparametric bootstrap replicates to assess nodal support. Nodes were considered strongly supported if their Bayesian posterior probability was ≥ 0.95 and their bootstrap value was ≥ 80 % (Huelsenbeck and Rannala 2004).

### Genetic distances

Finally, to obtain an estimate of genetic distances, pairwise genetic distances for COI were computed between and within the major clades obtained in the phylogenetic analysis (see results). The corrected pairwise genetic distances were calculated using the K2P model with MEGA version 7 (Kimura 1980, Kumar et al. 2016).

## Results

### Phylogenetic inferences

The results showed some incongruence between loci, but the major differences were between poorly-supported clades (Suppl. material 3–5). The final concatenated data set consisted of 1520 aligned nucleotide positions. The partitions and models that best fit the data were GTR+G (COI second positions, wg first and second positions, EF-1a third positions), and GTR+I+G (COI first and third positions, wg third positions, and EF-1a first and second positions). ML and Bayesian inference analyses resulted in highly congruent phylogenetic trees. The recovered relationships between the genera of Riodinidae were in agreement with recently published phylogenies (e.g., Espeland et al. 2015), thus providing a solid platform for the evaluation of the monophyly of *Apodemia*.

**Figure 1.**
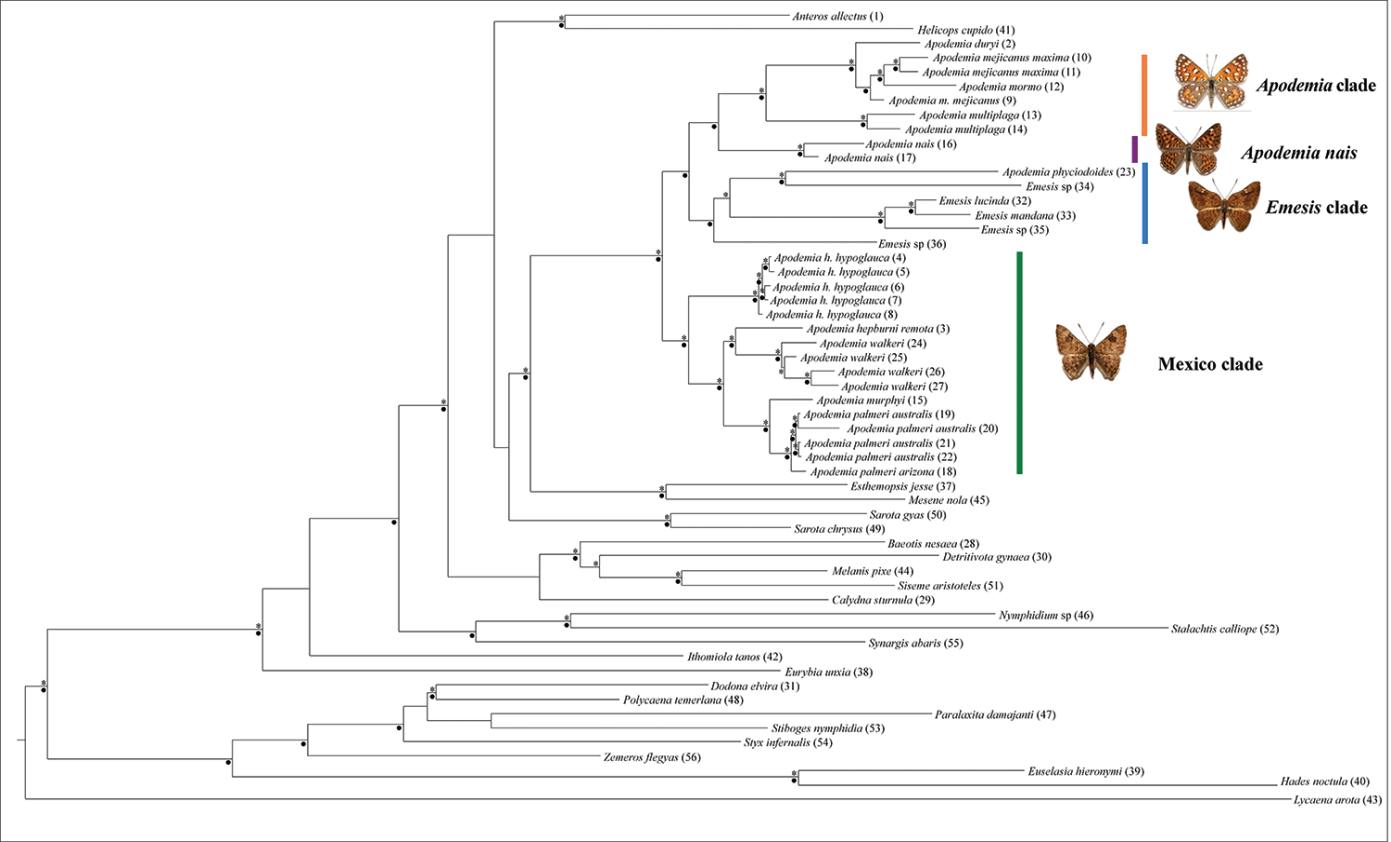
Phylogenetic relationships based on partial sequences of the mtDNA (COI), and nuclear genes (EF-1a, wg). Bayesian posterior probabilities and bootstrap values of major nodes are indicated with black dots for well-supported nodes. Vertical lines correspond to the major clades found in this study.

In the phylogenetic analyses, four clades can be distinguished (Fig. 1). The monophyly of *Apodemia* was not supported, as *Apodemia phyciodoides* W. Barnes & Benjamin was more related to *Emesis* than to other *Apodemia*. The rest of the species of *Apodemia* were included into three strongly supported clades. The first clade (*Apodemia* clade), included the North American taxa of the *A. mormo* complex: *A. mormo* and *A. mejicanus* (Behr), also *A. duryi* (W. H. Edwards) and *A. multiplaga* Schaus. The second clade included *A. nais*; and the third clade (Mexico clade) was composed by the *Apodemia* taxa distributed mostly in Mexico and Central America: *A. hepburni* Godman & Salvin, *A. hypoglauca* Godman & Salvin, *A. murphyi* Austin, *A. palmerii* W. H. Edwards and *A. walkeri* Godman & Salvin.

The relationships between the four major clades were as follows: the *Apodemia* clade was the sister group of *A. nais*, and this clade *Apodemia* + *A. nais* was the sister group of the clade *Emesis* + *A. phyciodoides* (*Emesis* clade). These relationships were strongly supported in the Bayesian analysis, but less supported in the ML analysis. The Mexico clade was the sister group of the rest of the species of *Apodemia* and *Emesis*.

Genetic distances within major clades ranged from 0.9% in *A. nais* to 9.8% in *Emesis* + *A. phyciodoides*, whereas distances between genera ranged from 7.4% in Mexico clade versus *A. nais* to 10.5% in the *Apodemia* clade versus *Emesis* clade. The genetic distance between *Apodemia* clade and Mexico clade was 8.7% (Table 1).

**Table 1.** Corrected pairwise genetics distances calculated with K2P model. Among (below diagonal) and within (diagonal) all major clades obtain in the phylogenetic analysis (see results) using only COI gene.

	*Apodemia* clade	Mexico clade	*A. nais*	*Emesis* clade
*Apodemia* clade	**0.055**			
Mexico clade	0.087	**0.050**		
*Apodemia nais*	0.081	0.074	**0.010**	
*Emesis* clade	0.104	0.095	0.093	**0.100**

### Descriptions of new genera

Diagnosis of *Plesioarida* Trujano-Ortega & García-Vázquez gen. n. and *Neoapodemia* Trujano-Ortega gen. n. are presented, comparing both with *Apodemia* (Table 2). The morphology of both genera is described, including photographs and illustrations of the structures; distribution maps of these new genera are also given. The extent of genetic divergence between them and the phylogenetic position within the Riodinidae are discussed. The morphologic examination of the specimens revealed that the genital structures of the species *A. phyciodoides*, *A. planeca*, and *A. castanea* are distinct from the other species of *Apodemia*; suggesting these are not congeneric with the *Apodemia* species of Central and North America. The inclusion of these three species in *Apodemia* is being evaluated (Seraphim et al. submitted, Trujano-Ortega unpublished data); therefore, the morphology of *Apodemia* reported here excludes these species.

**Table 2.** Comparison of selected morphological characters for the *Apodemia*, *Plesioarida*, and *Neoapodemia*.

Character	*Apodemia*	*Neoapodemia* gen. n.	*Plesioarida* gen. n.
**Labial palpus**			
Length of the first segment	Longer than the third segment	As long or longer than the third segment	As long or longer than the third segment
Length of the second segment	More than 2.5 the length of the first segment	Twice the length of the first segment	From 2 to 2.5 the length of the first segment
**Anterior wing**			
Vein Sc+R1 originates	in the second third of the discal cell^†^	in the last third of the discal cell	in the last third of the discal cell
**Prothoracic legs**			
Trochanter-coxa joint	Beyond half of the coxa	Beyond half of the coxa	At the middle of the coxa
Number of tarsomeres	Three tarsomeres	Three tarsomeres	Two tarsomeres ^‡^
Shape of the last tarsomere	Conic	Wide at the base, elongated and tapering toward the apex, with blunt end	Wide at the middle, oval-shaped, elongated, pointy at the end
Femur + trochanter length	Less than 3/4 the length of the tibia	More than 3/4 the length of the tibia	Less than 3/4 the length of the tibia
Tibia	Wider than the tarsus	Wider than the tarsus	As wide as the tarsus
**Male genitalia (lateral view)**			
Posterior margin of the uncus	Blunt	Blunt with a middle groove	Rounded
Tegumen	Wide	Wide	Narrow
Tegumen margins	Dorsal margin longer than anterior margin	Dorsal margin longer than anterior margin	Dorsal margin shorter than anterior margin
Posterior projection of the mid region of the vinculum hump-shaped	Evident, sclerotized	Evident, sclerotized	Less evident, slightly sclerotized
Length of the dorsal process of the valve	As long or shorter than the posterior margin of the uncus^§^	Shorter than the posterior margin of the uncus	Beyond the posterior margin of the uncus
Cornuti	Simple plate, long, strongly sclerotized	Multiple long spines, wide and sclerotized, jointed at the base (crest like), and flatten laterally	Multiple long spines, wide and sclerotized, in separated bulbs
**Host plant**			
	*Eriogonum* spp. (Polygonaceae) and *Krameria glandulosa* (Krameriaceae)	*Ceanothus fendleri* (Rhamnaceae)	*Prosopis* spp. and *Acacia* spp. (Fabaceae)
**Habitat**			
	Xerophile shrubland and Deciduous tropical forest	Coniferous forest	Xerophile shrubland, Deciduous tropical forest, and Evergreen tropical forest

^†^ Except in *A. multiplaga*; ^‡^ Except in *P. h. hypoglauca*; ^§^ Except in *A. multiplaga*.

#### Plesioarida

Taxon classificationAnimaliaLepidopteraRiodinidae

Trujano-Ortega & García-Vázquez gen. n.

http://zoobank.org/627AB6DD-9174-4175-A5AB-B6B34E586540

**Figure 2.**
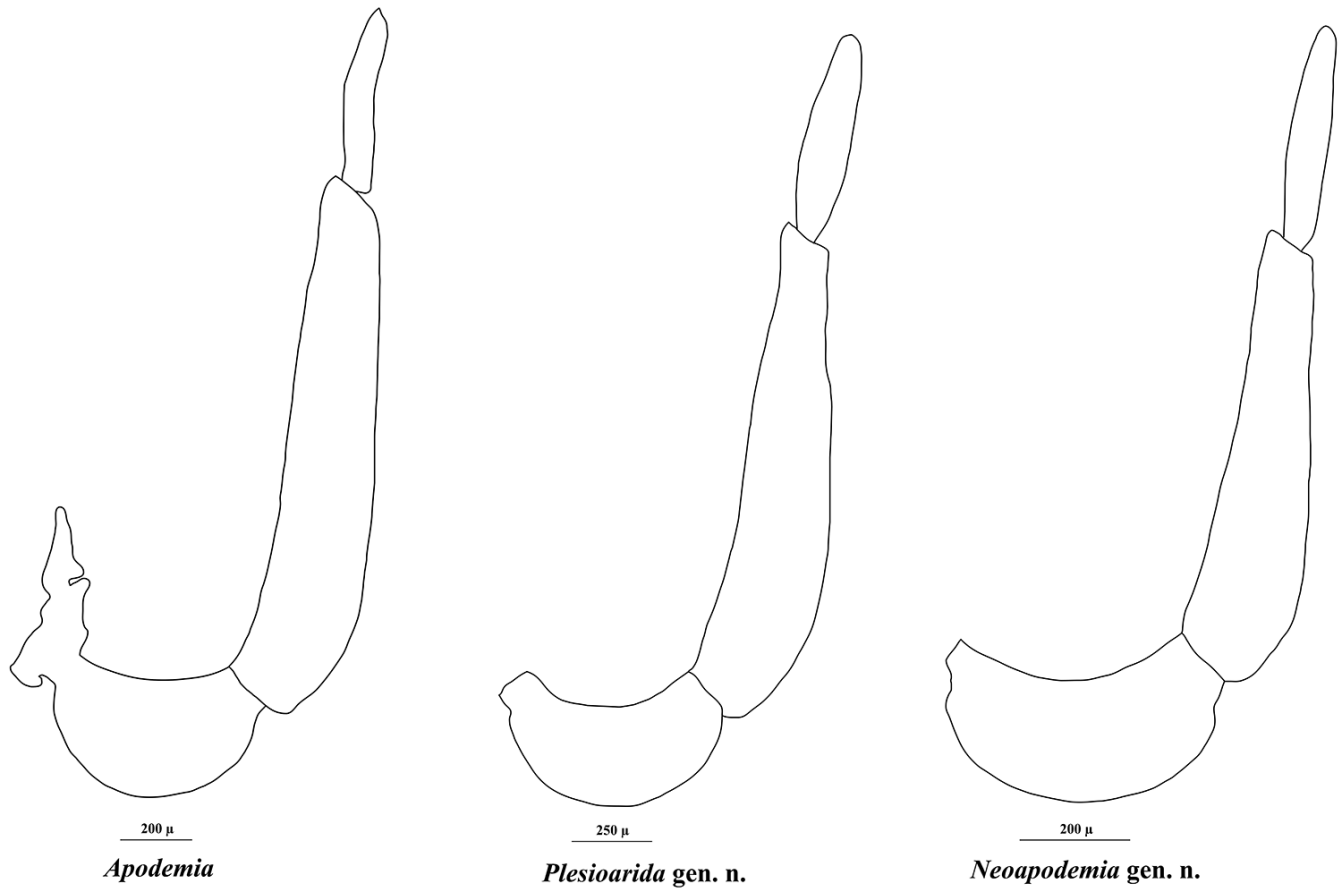
Left male palpus of *Apodemia*, *Plesioarida*, and *Neoapodemia*.

**Figure 3.**
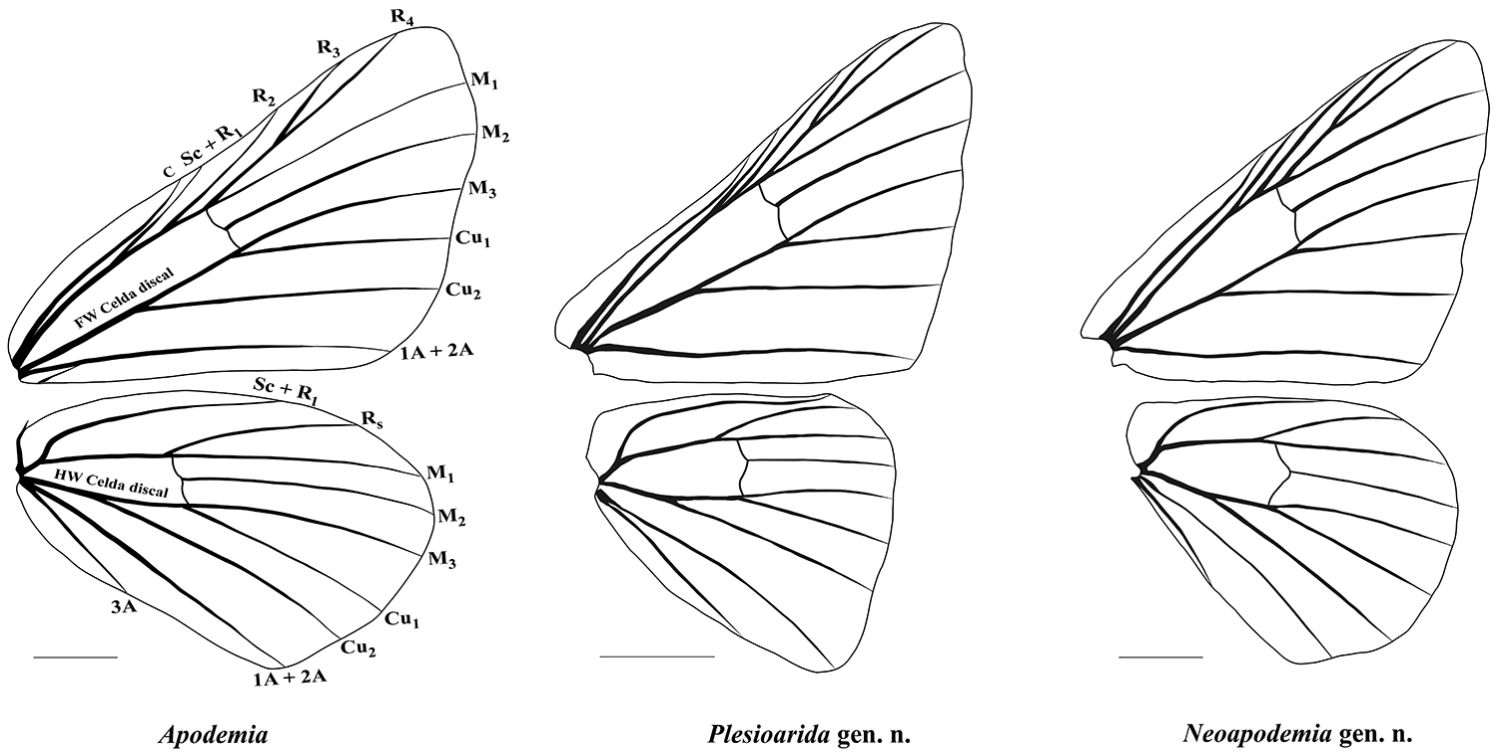
Wing venation of *Apodemia*, *Plesioarida*, and *Neoapodemia*. Upper, forewing; lower, hind wing. Vein abbreviations (black lettering): **Sc** subcostal, **R** radial, **M** median, **Cu** cubital, **A** anal. Scale bars: 3 mm.

**Figure 4.**
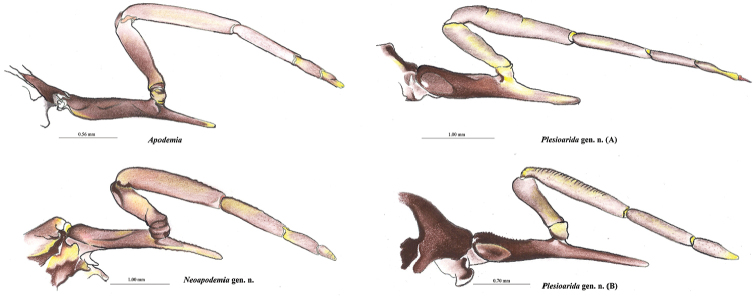
Prothoracic legs of males of *Apodemia*, *Plesioarida*, and *Neoapodemia*.

**Figure 5.**
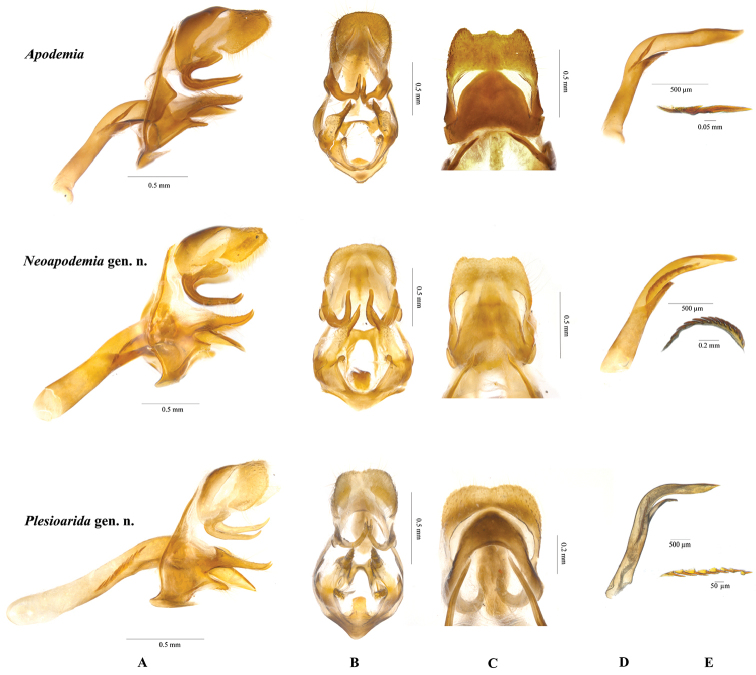
Male genitalia of *Apodemia*, *Plesioarida*, and *Neoapodemia.* **A** lateral view **B** ventral view **C** dorsal view **D** aedeagus **E** cornuti.

**Figure 6.**
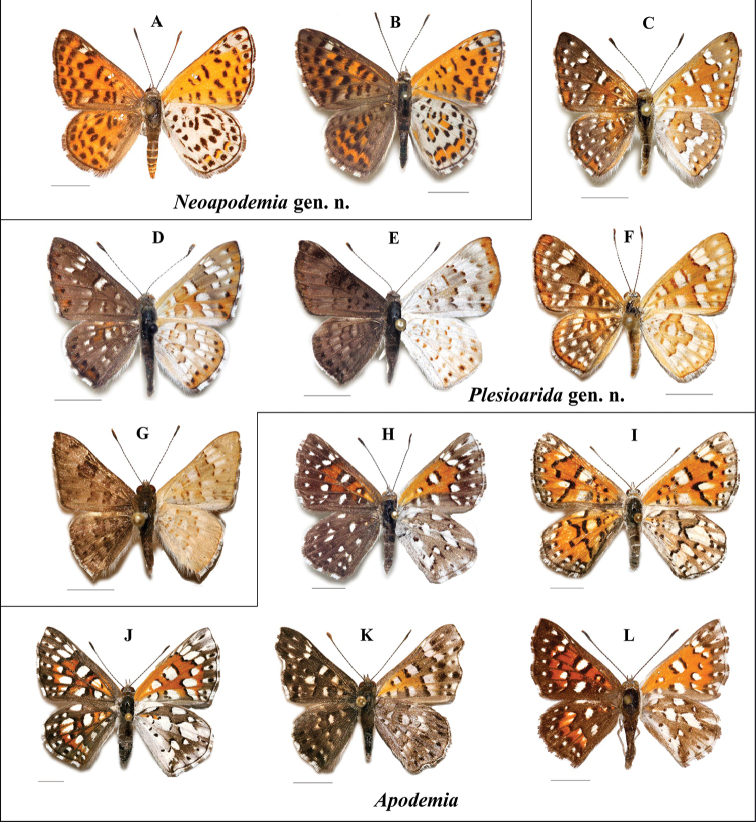
Wing color patterns of *Apodemia*, *Plesioarida* and *Neoapodemia* **A** *Neoapodemia chisosensis* comb. n. **B** *Neoapodemia nais* comb. n. **C** *Plesioarida murphyi* comb. n. **D** *Plesioarida h. hepburni* comb. n. **E** *Plesioarida h. hypoglauca* comb. n. **F** *Plesioarida p. palmerii* comb. n. **G** *Plesioarida walkeri* comb. n. **H** *Apodemia m. mormo* **I** *Apodemia duryi* **J** *Apodemia mejicanus* **K** *Apodemia multiplaga* **L** *Apodemia virgulti*. Scale bars: 5 mm. Additional data of the specimens in the photos are shown in Suppl. material 6.

Figs 2 , 3 , 4 , 5 , 6

##### Type species.

*Apodemia walkeri* Godman & Salvin, 1886 by present designation.

##### Diagnosis.

The species of this new genus can be distinguished from other Riodinidae by a combination of characters (Table 2). Labial palpi are long, slender, pointed apically and projected forward and upward, the second segment is a little more than twice the length of the third segment, the third segment is barely visible from dorsal view (Fig. 2). Radial veins originate near the end of the discal cell, costal vein runs parallel to Sc+R1; these veins get close but never fuse together. Vein R4 reaches wing margin at the apex (Fig. 3). Prothoracic legs of males are slender and trochanter inserts at the middle of the coxa; tibia is as wide as *tarsus* and smaller than the length of the femur plus the trochanter. Most of the species present two tarsomeres, the second tarsomere is oval-shaped and the apex pointed, except *P. hypoglauca* comb. n. which has three tarsomeres with the last one oval-shaped (Fig. 4). Tegumen of male genitalia is typically oval-shaped, narrow and slightly sclerotized, posterior half is a hyaline area that Hall (1999) named ‘windows’ through which the subescafium can be observed; the uncus is rounded and with setae in the posterior margin. The vinculum is a narrow band not covering the whole margin of the tegumen, is mostly straight and convex toward the saccus, a little hump-shaped in the mid region. Valvae are bifurcated, the dorsal process is conical, elongated, and with a sharp end projected forward and exceeding the posterior margin of the uncus, the ventral process is shorter and blunt with many setae. Aedeagus is long and sigmoid, wider in the anterior edge, slender and pointed on the posterior edge, where it opens dorsally. Cornuti are a series of wide, strongly sclerotized spines that originate from individual bulbs (Fig. 5).

##### Description.

**Male.** Anterior wing length: 10–15 mm. *Head.* Ringed antennae with 30 to 32 flagellomeres of the same width, with white scales at the base of each flagellomere. Widen abruptly in the apical 10 flagellomeres to form the antennal club, which is dark and iridescent dorsally. Sometimes white or brown scales are present at the sides, ending in a whitish or yellowish tip, with a nudum from flagellomere 20 to the apex. Labial palpi white with black or brown scales mainly in the third segment. *Wings* (Figs 3, 6) with four radial veins. Three distinct shapes of anterior wings, rounded toward the apex (*P. palmerii* comb. n.), elongated and triangular (*P. walkeri* comb. n.) and triangular with the external margin curved and the apex slightly sickle-like (*P. hypoglauca* comb. n.). Background color in both wings varies from brown to dark gray. Some species present a series of white spots outlined with black in the anterior margins and a series of submarginal black dots, sometimes with white scales and occasionally with reddish scales toward the base of the anterior and posterior wings. In grayish species spots are black. *Legs.* Prothoracic legs with dense long scales generally whitish, mid and hind legs with multiple short and dense spines in the interior margin of the tibia and *tarsus*. *Abdomen.* Dark in the dorsum with reddish or whitish scales outlining each segment. Ventrally with dense scales varying from whitish as in *P. hypoglauca* comb. n. to brown-orange as in *P. palmerii* comb. n. *Genitalia.* Genital capsule small, uncus rounded with a groove of variable depth which gives it a lobulated or straight appearance. Tegumen oval-shaped and sclerotized in the anterior region; with large ‘windows’ that reach the gnathi. Gnathi are slender, sclerotized, slightly twisted ending in an upward hook. Vinculum generally is straight or slightly curved near the tegumen, a little wider near the valve, this swelling is weakly sclerotized and hard to notice, curved before the saccus and anteriorly projected. Dorsal processes of the valve conic and membranous toward the transtilla but strongly sclerotized toward the apex, which is a small upward hook, with setae lengthwise. The ventral process is long and blunt, of variable lengths but always with multiple mostly long setae. Aedeagus is slender toward the distal portion with a pointed tip, widening toward the anterior portion, straight or sinuous. Cornuti are thick sclerotized spines apparently each surging from independent bulbs forming a line.

##### Etymology.

The name comes from the Greek *plesios* meaning near or close to and the Latin *aridus* meaning dry, in reference to the desert and semiarid habitats of most of the species.

##### Distribution and habitat.

**Figure 7.**
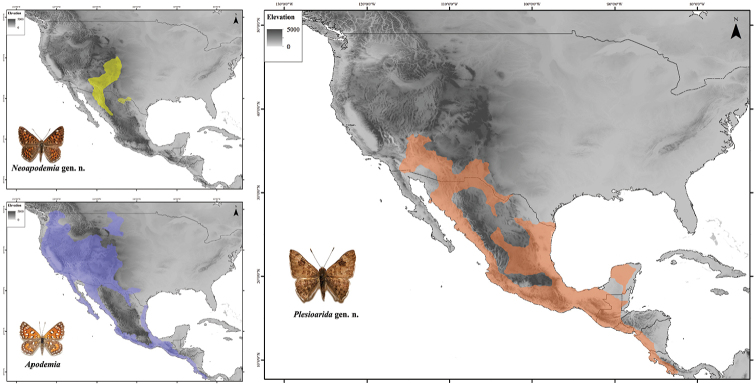
Known distribution of *Apodemia*, *Plesioarida*, and *Neoapodemia.* Black lines represent countries’ limits.

This genus is distributed below 1750 m in the Pacific slope from central Arizona and in the Atlantic slope from the south of Texas to the dry forests of Guanacaste in the northeast of Costa Rica (DeVries 1997) (Fig. 7). In the USA, it has been collected in arid regions, in xerophilic shrubland of Arizona, California, and New Mexico, with isolated records in the south of Texas in Río Grande Valley (Warren et al. 2017). In Mexico it can be found in deserts and semiarid regions of Baja California Sur and part of Baja California Norte in the Chihuahuan Desert and in the Mexican Plateau, regions were xerophilic shrubland is dominant. It is also distributed in the deciduous tropical forest of the west of Mexico in the Pacific coast and the Balsas Basin, as well as in the east in the coastal plain of the Gulf of Mexico and the Yucatan Peninsula. Its distribution in deciduous tropical forests extends through Central America to Costa Rica. Finally, *P. selvatica* comb. n. and some populations of *P. walkeri* comb. n. inhabit the tropical forests in the south of Mexico in Veracruz and Chiapas. De la Maza and De la Maza (2017b) mention that *P. selvatica* comb. n. probably is present in Guatemala and Belize.

##### Natural history.

Larvae of the species of *Plesioarida* Trujano-Ortega & García-Vázquez gen. n. are associated with the family Fabaceae, particularly with species of the genera *Prosopis* spp. and *Acacia* spp. (Ferris 1985, Austin 1988, DeVries 1997).

### A proposed classification of *Plesioarida* gen. n.

*Plesioarida* Trujano-Ortega & García-Vázquez, gen. n. Type species: *Apodemia walkeri* Godman & Salvin, [1886], Biol. Centr. Amer., Lepid. Rhop. 1(45): 468, no. 6, by present designation.

***hepburni*** comb. n. (*Apodemia*).

*Apodemia hepburni* Godman & Salvin, 1886. Type Locality: “Mexico, Pinos Altos in Chihuahua”.

*h. hepburni* Godman & Salvin, 1886, comb. n. (*Apodemia*).

*h. remota* Austin, 1991, comb. n. (*Apodemia*). Type Locality: “México: Baja California Sur; Arroyo San Bartolo”.

***hypoglauca*** comb. n. (*Apodemia*).

*Lemonias hypoglauca* Godman & Salvin, 1878. Type Locality: “Mexico”.

*h. hypoglauca* Godman & Salvin, 1878, comb. n. (*Apodemia*).

*h. wellingi* Ferris, 1985, comb. n. (*Apodemia*). Type Locality: “México: Yucatán; Pisté”.

***murphyi*** comb. n. (*Apodemia*).

*Apodemia murphyi* Austin, [1989]. Type Locality: “México: Baja California Sur; Arroyo San Bartolo”.

***palmerii*** comb. n. (*Apodemia*).

*Lemonias palmerii* Edwards, 1870. Type Locality: “Utah”

*p. palmerii* W. H. Edwards, 1870

= *p. marginalis* Skinner, 1920

*p. arizona* Austin, [1989], comb. n. (*Apodemia*). Type Locality: “Arizona: Cochise County, Arizona State Route 90, 10.8 miles north of Arizona State Route 82”

*p. australis* Austin, [1989], comb. n. (*Apodemia*). Type Locality: “México: Durango: 1 mi. S Nombre de Dios”.

***selvatica*** comb. n. (*Apodemia*).

*Apodemia selvatica* De la Maza & De la Maza, 2017. Type Locality: "Estación Chajul" [México, Chiapas].

***walkeri*** comb. n. (*Apodemia*).

*Apodemia walkeri* Godman & Salvin, 1886. Type Locality: “Mexico, Acapulco” [Guerrero]

#### Neoapodemia

Taxon classificationAnimaliaLepidopteraRiodinidae

Trujano-Ortega gen. n.

http://zoobank.org/749DBAD0-54D3-4AA0-8095-704228745AD0

Figs 2 , 3 , 4 , 5 , 6

##### Type species.

*Chrysophanus nais* Edwards, 1876 by present designation.

The original generic name for *Apodemia nais*, *Chrysophanus* Hübner, 1818, is unavailable, having been suppressed by the ICZN. In 1886, Godman and Salvin proposed the generic name *Polystigma*, with *Chrysophanus nais* W.H. Edwards as its type species. However, *Polystigma* Godman & Salvin, 1886 is invalid, being a junior homonym of *Polystigma* Kraatz, 1880 (Coleoptera). Therefore, we propose the name *Neoapodemia* for this taxon.

##### Diagnosis.

This genus contains two species that can be distinguished from other Riodinidae by the presence of the labial palpi that are medium sized, slender, sharp apically and projected upward and forward; the second segment is twice the length of the third segment; third segment and apical third of the second segment are visible from dorsal view (Fig. 2, Table 2). Radial veins originate near the end of the discal cell, costal vein parallel to Sc+R1 separated by at least twice the width of R1 in the closest portion. Vein R4 reaches de margin in the wing apex (Fig. 3). Prothoracic legs of male are slender, trochanter joint beyond the middle of the coxa, tibiae are slightly wider than the tarsi and the length of the femur + trochanter is nearly the same as tibia length. Tarsus with three tarsomeres, the third tarsomere is small, wide at the base tapering toward the apex but with a blunt end (Fig. 4). Male genitalia with tegumen typically oval-shaped, wide, strongly sclerotized in the dorsal region, posterior half hyaline; the uncus is rectangular with a groove in the distal margin, with setae. Gnathi are wide, curved and ending in a sharp tip projected dorsally. Vinculum is a wide sclerotized band not covering the whole margin of the tegumen, generally straight and convex toward the saccus, with an evident swallowing in the mid region. Valvae bifurcated, dorsal process conical of variable width and setae all along, ending in a sharp tip projected dorsally, this process is shorter than the posterior edge of the uncus; the ventral process is short and with a rounded tip, projected anteroventrally, also with setae. Cornuti are a series of long, flatten laterally, strongly sclerotized spines, joined at the base in a crest-like shape (Fig. 5).

##### Description.

**Male.** Anterior wing length: 15–18 mm. *Head*. Ringed antennae with 40 flagellomeres with white scales at the base of each flagellomere. Widen in the apical 18–22 flagellomeres, where a nudum is present and it extends to the apex ending in a whitish or yellowish tip. The antennal club is formed by the apical 10–12 flagellomeres, black dorsally and sometimes with a line of white scales. Labial palpi white with black or brown scales in the third segment.

*Wings* (Figs 3, 6). Triangular with four radial veins. In dorsal view, background color varies in both wings from brown to copper-orange, margins are darker and costal and external margins are outlined with black. External margin with a line of seven rounded dots and a line of rectangular spots in the submarginal area which cross the wing from the costal to the anal margin in both wings. Three subapical white spots, the first two are just short lines and the third is squared and large, situated in the R4 cell. After the spots is an irregular band of black spots in the postmedian area going into the median area. Discal cell with four black bands, with the most external one larger and wider than the remainder. Under the discal cell are three other bands in the postbasal and submedian areas. White and black fringe present in diverse patterns. Particularly, *N. nais* comb. n. presents copper-orange scales in the discal cell and between the lines of black spots on both wings. Ventrally, anterior wing is orange, lighter and brighter than dorsal view, with the same pattern of black spot as in dorsal side, white spot of R4 cell extends till the apex; the black dots forming the marginal line are surrounded by white scales, as the dots approach the tornus the white scales are present only in the posterior margin. The posterior wing is white at the base and with essentially the same pattern of black spots as in dorsal view, however the base of *N. nais* comb. n. is white-greyish and the white scales over the veins provide a less uniform pattern to the wing than in *N. chisosensis* comb. n. This species presents three orange spots in the posterior margin of the line of submarginal spots, while in *N. nais* comb. n. the orange area is between the line of black submarginal and marginal spots, getting wider as it approaches the tornus. This species also presents an orange area along the costal margin just before the apex and another one in the anterior margin of the irregular band of black spots in the median area.

*Legs.* Prothoracic legs have dense and long scales generally white or whitish; mid and hind tibiae and tarsi with a series of multiple short and dense white, whitish or yellowish spines in the inner margin.

*Abdomen.* Dorsum of abdomen dark of brown with orange and whitish scales outlining each segment. Ventrally, the abdomen is bright white in *N. chisosensis* comb. n. and whitish in *N. nais* comb. n.

*Genitalia.* Generally strongly sclerotized, genital capsule medium sized. The margin of the uncus in dorsal view presents great variation, it can be rounded or straight with a groove of variable depth. Dorsal processes of the valve are conic and membranous toward the transtilla but narrower in *N. nais* comb. n. than in *N. chisosensis* comb. n.; the ventral process is also narrower and longer in *N. nais* comb. n. Aedeagus of *N. nais* comb. n. is wide, short and sclerotized, of uniform width all along, with a more sclerotized plate in the dorsum and ending in a sharp tip in the posterior edge where it opens dorsally. In *N. chisosensis* comb. n. aedeagus is narrower, longer and less curved, it slightly widens in the anterior edge and makes narrow toward the posterior edge, with a more sclerotized dorsal plate ending in a blunt tip. Cornuti are a series of long, flattened laterally, strongly sclerotized spines, joined at the base in a crest-like shape (Fig. 5E).

##### Etymology.

The name is a combination of the Greek prefix *neo*, meaning new, and *Apodemia*, in reference to the genus from which it separates.

##### Distribution and habitat.

This genus has a disjunct distribution. *Neoapodemia nais* comb. n. is distributed in montane areas with medium to high elevations (1600–2300 m), mostly in the southern and southwestern Rocky Mountains in the USA. In USA inhabits chaparral and open areas of coniferous forests in northern and central Colorado, southeastern New Mexico, and central and southeastern of Arizona where its presence appears to be sporadic (Scott 1986, Brock and Kaufman 2006). In Mexico it can be found in the Sierra Madre Occidental in the states of Sonora, Chihuahua, and Durango (Warren et al. 2017). On the other hand, the distribution of *N. chisosensis* comb. n. is restricted to western Texas in the Chisos Mountains in Big Bend National Park, where it inhabits the chaparral of submontane shrubland.

##### Natural history.

Larvae of *Neoapodemia* Trujano-Ortega, gen. n. can be found feeding on plants of the Rosaceae family, *Prunus havardii* (W. Wight) S.C. Mason, and the Rhamnaceae, *Ceanothus fendleri* A. Gray (Scott 1986, Brock and Kaufman 2006, Warren et al. 2017).

### A proposed classification of *Neoapodemia* gen. n.

*Neoapodemia* Trujano-Ortega, gen. n. Type species: *Chrysophanus nais* W. H. Edwards, 1876, Trans. Am. Entomol. Soc. 5(3/4): 291–292, by present designation.

= *Polystigma* Godman & Salvin, [1886]. Biol. Centr. Amer., Lepid. Rhop. 1(45): 469. Type-species: *Chrysophanus nais* W. H. Edwards, 1876, Trans. Am. Entomol. Soc. 5(3/4): 291–292, by monotypy. Preoccupied by *Polystigma* Kraatz, 1880, Dtsche. Entomol. Z. 24(2): 191.

***nais*** comb. n. (*Apodemia*).

*Chrysophanus nais* W. H. Edwards, 1876. Type Locality: “Southern California... Prescott, Arizona”.

***chisosensis*** comb. n. (*Apodemia*).

*Apodemia chisosensis* Freeman, 1964. Type Locality: “Chisos Mountains, elevation 5400 ft., Texas”

### Taxonomic remarks

The phylogenetic analysis based on molecular data shows three well-supported clades which are also distinguished by their morphology. The clade *Neoapodemia* is more related to the clade of *Apodemia* (*sensu stricto* excluding *A. castanea* and *A. phyciodoides*) than with *Plesioarida*. Besides the phylogenetic position, the proposed genera can be easily distinguished morphologically as well as with *Apodemia* and *Emesis*. Labial palpi of *Apodemia*, *Plesioarida* and *Neoapodemia* are slender, long and projected upward and forward, while *Emesis* present small labial palpi, close to the head and directed upward. The second segment of labial palpi in *Neoapodemia* is smaller in proportion with the third segment and only one third is visible in dorsal view, in contrast with *Apodemia* in which the second segment is long and half or more of it is visible in dorsal view. *Plesioarida* differs from both genera having only part of the third segment visible in dorsal view. Regarding wing veins patterns, radial veins of *Neoapodemia* and *Plesioarida* originate near the end of the discal cell, whereas in *Apodemia* these veins originate at the middle of the discal cell. Veins C and Sc+R1 are closer in *Plesioarida* and *Apodemia* than in *Neoapodemia*, which present a clear separation between these veins. The number and shape of the tarsomeres of prothoracic legs are useful characters for separating the genera. *Emesis* has only one tarsomere, *Plesioarida* has two (except *P. hypoglauca* comb. n. which has three) and an oval-shaped tip, *Neoapodemia* and *Apodemia* presents three tarsomeres; the last tarsomere in *Apodemia* is conical, while *Neapodemia* the last tarsomere is smaller than in *Plesioarida*, wide at the base, tapering toward the apex and ending in a blunt tip.

*Apodemia* and *Neoapodemia* present large genital capsules, more similar than with those of *Plesioarida*. However, *Neoapodemia* gnathi are slightly twisted and the posterior tip is hooked and strongly projected upward; while gnathi of *Apodemia* are straight. The genital capsule of *Plesioarida* is smaller and rounded, with tegumen with a marked hyaline area; gnathi are slim and twisted. Vinculum is straight in *Apodemia*; convex in *Neoapodemia*, which makes it appear larger; and straight near tegumen and convex toward the saccus in *Plesioarida*. Dorsal processes of *Plesioarida* differ from the other two genera because are long and exceed the posterior margin of the uncus. The ventral processes of the valves of *Neopapodemia* are shorter than those of *Apodemia* and *Plesioarida*, which are also slender. Aedeagus bends in a smooth angle in *Neoapodemia*, but in a marked angle in *Plesioarida*, in both genera this bending appears near the posterior tip of the aedeagus. In *Apodemia* the bending angle is marked (approximately 45°), nearly at half of aedeagus or closer to the anterior tip. The cornuti are one of the most important differences. The cornuti of *Apodemia* are simple, long, and sclerotized; *Plesioarida* presents a series of aligned spines that surge from individual bulbs; finally, *Neoapodemia* has long, strong, sclerotized spines, flattened laterally and joined at the base, forming a crest. *Emesis* genitalia are diverse and the cornuti are simple when present.

The phylogenetic analysis with molecular data suggests that *A. phyciodoides* is part of *Emesis*. However, we took a conservative approach and decided that *A. phyciodoides* should remain in *Apodemia* until morphology was reviewed. Considering that *Emesis* need a taxonomical review and that the *Emesis* + *A. phyciodoides* clade was well supported only by Bayesian but not ML method.

Natural history characters also help to distinguish between these groups. For example, it is noteworthy that each genus has distinct host plants (Ferris 1985, Scott 1986, Austin 1988, DeVries 1997, Brock and Kaufman 2006, Warren et al. 2017), which is evidence of differences in their life histories. Although none of these genera is exclusive of an environment, some of their species are characteristic of specific habitats. For example, *Apodemia* inhabits desert areas and shrubland environments (although *A. multiplaga* is from deciduous tropical forests). *Neoapodemia* is recorded from coniferous forest, chaparral, and submontane shrublands. *Plesioarida* inhabits desert areas and deciduous tropical forests.

### Conclusions

Our results show that *Apodemia* is paraphyletic with respect to *Emesis*; furthermore, the morphological evidence and the preliminary molecular analysis (Trujano-Ortega unpublished data) suggest polyphyly. This is because, historically, diverse unrelated lineages have been placed within *Apodemia* and reassigned posteriorly. Harvey and Clench (1980) created the genus *Dianesia* for *Apodemia carteri* and suggested that *A. castanea* is not congeneric with North and Central American *Apodemia*. More recently, Penz and DeVries (2006) reassigned *Apodemia paucipuncta* to the recently described genus *Hallonympha*.

In a previous analysis of the phylogenetic relations of Riodinidae at the tribe or subfamily level, *Apodemia* and *Emesis* appear always closely related based on morphological evidence (Stichel 1910–1911, Harvey 1987) and molecular evidence (Saunders 2010, Espeland et al. 2015, Proshek et al. 2015). These two genera were grouped in the same tribe Emesini of Stichel (1910–1911). However, the taxonomic scale of these studies and the lack of recent material in scientific collections limited the resolution of lower taxonomic scale and the inclusion of Mexican and Central American species. That is why the two genera proposed here remained unnoticed. The morphological characters shared by *Apodemia*, *Emesis*, *Neoapodemia*, and *Plesioarida* are evident, as suggested by Harvey (1987), who analyzed the immature stages (pupae). These genera are more related to each other than with the genera of any other tribe of Riodinidae. Therefore, these two new genera should be included in a new tribe as mentioned by Espeland et al. (2015) for several *Incertae sedis* groups, like *Emesis*-*Apodemia*. The biogeographic patterns of these new genera are an interesting topic for further study. Espeland et al. (2015) proposed two independent dispersal events for these genera, from the Neotropical to the Nearctic region. One event of the ancestor of *Apodemia* mainly to the southwestern USA and the other event of the ancestor of *Calephelis*, to the eastern and central USA.

We consider that each of these genera is well supported by the morphological and molecular evidence. In order to resolve these phylogenetic relations a more extensive sampling is required, joined with a detailed review of the morphology of most species, including *Emesis* species. Also, the species *A. phyciodoides*, *A. castanea*, and *A. planeca* must be assigned to the correct genera in order to stabilize *Apodemia*. This can only be achieved with the use of diverse character systems and sufficient sampling. This study contributes to the systematics and classification of Riodinidae. It adds two genera to the family: *Neoapodemia* which, as *Apodemia*, is exclusive to North America, and *Plesioarida* of North and Central America.

## Supplementary Material

XML Treatment for Plesioarida

XML Treatment for Neoapodemia

## Supplementary materials

Supplementary material 1 Specimens examined for morphological revision Data type: MS Word file. Explanation note: Location data for specimens. This is an open access article distributed under the terms of the Creative Commons Attribution License (CC BY 4.0), which permits unrestricted use, distribution, and reproduction in any medium, provided the original author and source are credited.Marysol Trujano-Ortega, Uri Omar García-Vázquez, Curtis J. Callaghan, Omar Ávalos-Hernández, Moisés Armando Luis-Martínez, Jorge Enrique Llorente-Bousquets

Supplementary material 2 Collection and voucher data for Riodinids genetic samples used in this study Data type: MS Word file. Explanation note: Voucher data and GenBank accession numbers of samples used in the molecular analyses. The table is arranged alphabetically by species name and voucher number. This is an open access article distributed under the terms of the Creative Commons Attribution License (CC BY 4.0), which permits unrestricted use, distribution, and reproduction in any medium, provided the original author and source are credited.Marysol Trujano-Ortega, Uri Omar García-Vázquez, Curtis J. Callaghan, Omar Ávalos-Hernández, Moisés Armando Luis-Martínez, Jorge Enrique Llorente-Bousquets

Supplementary material 3 Maximum likelihood tree of the relationships among *Apodemia* and selected species in the Riodinidae inferred with Cytochrome Oxidase I (COI). Numbers near branch nodes are bootstrap branch support Data type: Molecular data. Explanation note: Tree infered with Cytochrome Oxidase I gene. This is an open access article distributed under the terms of the Creative Commons Attribution License (CC BY 4.0), which permits unrestricted use, distribution, and reproduction in any medium, provided the original author and source are credited.Marysol Trujano-Ortega, Uri Omar García-Vázquez, Curtis J. Callaghan, Omar Ávalos-Hernández, Moisés Armando Luis-Martínez, Jorge Enrique Llorente-Bousquets

Supplementary material 4 Maximum likelihood tree of the relationships among *Apodemia* and selected species in the Riodinidae inferred with Wingless (wg). Numbers near branch nodes are bootstrap branch support Data type: Molecular data. Explanation note: Tree infered with Wingless gene. This is an open access article distributed under the terms of the Creative Commons Attribution License (CC BY 4.0), which permits unrestricted use, distribution, and reproduction in any medium, provided the original author and source are credited.Marysol Trujano-Ortega, Uri Omar García-Vázquez, Curtis J. Callaghan, Omar Ávalos-Hernández, Moisés Armando Luis-Martínez, Jorge Enrique Llorente-Bousquets

Supplementary material 5 Maximum likelihood tree of the relationships among *Apodemia* and selected species in the Riodinidae inferred with gene Elongation factor 1 α (EF-1a). Numbers near branch nodes are bootstrap branch support Data type: Molecular data. Explanation note: Tree infered with Elongation factor 1 α gene. This is an open access article distributed under the terms of the Creative Commons Attribution License (CC BY 4.0), which permits unrestricted use, distribution, and reproduction in any medium, provided the original author and source are credited.Marysol Trujano-Ortega, Uri Omar García-Vázquez, Curtis J. Callaghan, Omar Ávalos-Hernández, Moisés Armando Luis-Martínez, Jorge Enrique Llorente-Bousquets

Supplementary material 6 Data of the photographs shown in the Figure 6 Data type: MS Word file. Explanation note: Location data and credits for the images. This is an open access article distributed under the terms of the Creative Commons Attribution License (CC BY 4.0), which permits unrestricted use, distribution, and reproduction in any medium, provided the original author and source are credited.Marysol Trujano-Ortega, Uri Omar García-Vázquez, Curtis J. Callaghan, Omar Ávalos-Hernández, Moisés Armando Luis-Martínez, Jorge Enrique Llorente-Bousquets
