# Supplementary material for: Two new genera of metalmark butterflies of North and Central America (Lepidoptera, Riodinidae)
Source: Zookeys. 2018 Jan 16;(729):61–85. doi: 10.3897/zookeys.729.20179 (PMC5799793; doi:10.3897/zookeys.729.20179)
Supplement: Supplementary material 1 — Specimens examined for morphological revision [file zookeys-729-061-s001.docx]

**Supplement file SF 1. Specimens examined for morphological revision**

Institutional codes for scientific collections: MZFC LEP = Colección Lepidopterológica del Museo de Zoología, Facultad de Ciencias, UNAM; MZFZ = Colección Lepidopterológica del Museo de Zoología, FES Zaragoza, UNAM; CNIN-IBUNAM = Colección Nacional de Insectos, Instituto de Biología, UNAM; MGCL = McGuire Center for Lepidoptera and Biodiversity, Florida Museum of Natural History, University of Florida, USA and CJC Collection of Curtis Callaghan, Colombia. ABD, MTO and BCS are field identifiers for uncatalogued specimens being deposited in the MZFC. Data are arranged alpha­betically by species name and geographic range in north/south order.

***Apodemia castanea***. **Brasil: Minas Gerais (1):** Km 68 Piamontina Carmeloso, 1♂ 5/IV/1975 leg (CJC uncataloged).

***Apodemia duryi*. México: Coahuila (2):** Cuatro Ciénegas, Ejido El Oso, (27.05938 N, 102.22661 W), 1151 m, 2 ♂♂ (MZFC LEP 224300, 414855), 04/X/2012 leg. M. Trujano-Ortega. **Querétaro (1):** 10 mi of Calinda, 1 ♂ (MZFC LEP, uncataloged), 28/V/1982, leg. J. Kemner.

***Apodemia mejicanus.* México: Baja California Sur (6):** Mulegé, 4.3 km S Mulegé, Hotel Serenidad (26.89132 N, 111.95501 W), 1 m, 1 ♂ (MTO 555) 07/IV/2014 leg. M. Trujano-Ortega, U. García; Loreto, 3 km SW de Loreto (25.99203 N, 111.35838 W), 7 m, 5 ♂♂ (BCS 45, 51, 52, 54, 58) 07/IV/2014 leg. M. Trujano-Ortega, U. García.

***Apodemia mormo.* México: Baja California (2):** Mexicali, Mis. San Catarina, North district Baja California, 2 ♂♂ (CNIN-IBUNAM 27772, 27773) 08/IV/1982 leg. M. F. M. Brown, David K. Faulkner. **USA: California (1):** Fresno, Parkfield Grade Road 8.0 road miles S. of CA 198, 1 ♂ (MGCL FL19), 3000 ft 05/IX/2001.

***Apodemia multiplaga.* México: Oaxaca (1):** Santa María Huatulco, Pluma Hidalgo, Rancho Hagia Sofía, 1 ♂ (MZFC LEP 378850) 03/VI/2013 leg. J. L. Salinas. **Guerrero (1):** Piedras Negras, Pilcaya, 1 ♂ (MZFZ 8149) 05/X/2012 leg. A. Ramírez G.

***Apodemia planeca*. México: Michoacán (4):** Múgica, El Marqués, 3 ♂ (MZFC LEP 120573–120576), 23/III/1997, 500 m.

***Apodemia virgulti.* México: Baja California (2):** Tijuana, Tecate, 8 mi E, Hwy. 2, 2 ♂♂ (CNIN-IBUNAM 27757, 27758) 25/V/1980 and 9/V/1980 leg. M. F. M. Brown, David K. Faulkner.

***Plesioarida hepburni.* México: Baja California Sur (6):** La Laguna, San Bartolo, (23.73592 N, 109.84294 W), 4 ♂♂ (MTO 496, MTO 509–11), 375 m, 03/IV/2014, leg. M. Trujano-Ortega and U. García; Los Cabos, Caduaño, 1♂ (CNIN-IBUNAM 27236), 22/I/1961; Los Cabos, Migriño, (23.04835 N, 110.097 W), 1 ♂ (MTO 528), 15 m, 5/V/2015, leg. M. Trujano-Ortega and U. García. **Sinaloa (1):** Sibajahui, El Fuerte (17.34583 N, 99.44777 W), 45 m, 1 ♂ (MTO 643) 9/XII/2014, leg. M. Trujano-Ortega. **Sonora (1)**: Huásabas (29.9219 N, 109.296 W), 1♂ (MTO 665), 510 m, 11/VII/2014, M. Trujano-Ortega, U. García, A. Arellano.

***Plesioarida hypoglauca.* México: Guerrero (4):** Acahuizotla, Chilpancingo de los Bravo, 1 ♂ (CNIN-IBUNAM 42327) VII/1992 leg. A. Ibarra; Buena Vista de Cuellar, Los Amates, 1 ♂ (MZFZ 7088) 08/IX/2012 leg. M. Luna; Ixcateopan de Cuauhtémoc (18.4943 N, 99.7889 W), 1727 m, 1 ♂ (MTO 392, 397), 17/X/2013, leg. A. Arellano. **Yucatán (1):** Ebtún, Valladolid (20.66359 N, 88.26498 W), 43 m, 2 ♂♂ (MTO 949, 950), 27/XIII/2014, leg. M. Trujano-Ortega, U. García. **Sinaloa (1)**: Aserradero, El Palmito (23.5564 N, 105.8833 W), 1827 m, 1 ♂ (MTO 335), 05/VII/2013, leg. M. Trujano-Ortega, U. García, A. Arellano. **Aguascalientes (1)**: Road Mex. 45, Aguascalientes/León, km 116 (21.7732 N, 102.278 W), 1877m, 1 ♂ (MTO 857), 20/VII/2014, leg. M. Trujano-Ortega, U. García, A. Arellano. **Querétaro (1)**: Cadereyta de Montes, Cadereyta de Montes (20.70951 N, 99.8515 W), 2011 m, 1 ♂ (MTO 1209), 17/XI/2014, leg. M. Trujano-Ortega, A. Arellano, O. Ávalos, U. García.

***Plesioarida murphyi.* México: Baja California Sur (6):** Santa Rita, 7.4 km SW de Santa Rita, Carr. Santa Rita/Puerto Chale (24.53879 N, 111.50282 W), 24 m, 2 ♂♂ (BCS 12–13) 08/IV/2014, leg. M. Trujano-Ortega, U. García; La Paz, 300 m S CIBNOR (24.133 N, 110.4252 W), 24 m, 1 ♂ (BCS 99), 10/IV/2014, leg. M. Trujano-Ortega, U. García; Los Cabos, El Migriño (23.04835 N, 110.09744 W), 14 m, 1 ♂ (MTO 529), 15/IV/2014, leg. M. Trujano-Ortega, U. García; Loreto, 3 km SW de Loreto (25.992 N, 111.3584 W), 7 m, 1 ♂ (MTO 541), 6/IV/20/2014; La Laguna, San Bartolo, (23.73592 N, 109.84294 W), 1 ♂ (MTO 513), 375 m, 03/IV/2014, leg. M. Trujano-Ortega and U. García.

***Plesioarida palmerii.* México: Querétaro (2)**: Cadereyta de Montes, Cadereyta de Montes (20.70951 N, 99.8515 W), 2011 m, 1 ♂ (MTO 1209), 17/XI/2014, leg. M. Trujano-Ortega, A. Arellano, O. Ávalos, U. García; Peña Miller, San Juanico (21.061 N, 98.1983 W), 1363 m, 1 ♂ (MTO 1204), 16/XI/2014, leg. M. Trujano-Ortega, A. Arellano, O. Ávalos, U. García. **Zacatecas (1)**: Carretera 49, Río Grande-Fresnillo, km 53 (23.7733 N, 103.011 W), 1944 m, 1 ♂ (MTO 822), 18/VII/2014, leg. M. Trujano-Ortega, A. Arellano, U. García. **Sinaloa (2)**: El Fuerte, Sibajahui (26.1632 N, 108.746 W), 45 m, 1 ♂ (MTO 643), 09/VII/2014, leg. M. Trujano-Ortega, A. Arellano, U. García; Ahome, Topoviejo, El Maviry (25.6005 N, 109.096 W), 16 m, 1 ♂ (MTO 468), 25/III/2014, leg. M. Trujano-Ortega, A. Arellano, U. García. **Durango (2)**: Nombre de Dios (23.8561 N, 103.7449 W), 1780 m, 2 ♂♂ (MTO 302, 303), 3/VIII/2013, leg. M. Trujano-Ortega, A. Arellano, J. Hernández, U. García. **Nuevo León (1)**: Dr. Arroyo (23.6993 N, 99.7394 W), 1882 m, 1 ♂ (MTO 238), 1/VII/2013, leg. M. Trujano-Ortega, A. Arellano, J. Hernández, U. García. **Coahuila (1)**: Cuatro Ciénegas de Carranza, Rancho Orozco, Poza Tío Cándido (26.8696 N, 102.079 W), 720 m, 1 ♂ (MTO 214), 5/X/2012, leg. M. Trujano-Ortega. **Sonora (1)**: Huásabas (29.9219 N, 109.296 W), 1♂ (MTO 663), 510 m, 11/VII/2014, M. Trujano-Ortega, U. García, A. Arellano. **USA: Nevada (1):** Clark, E. side of Bowman Reservoir, near Logandale, 1300 ft, 1 ♂ (MGCL FL12), 18/V/1998.

***Plesioarida walkeri.* México: Guerrero (1)**: Huitzuco, Agua Salada, 1 ♂ (MZFZ 7750) 04/X/2012 leg. M. Luna. **Morelos (5)**: Huexca, Road Cuautla-Huexca, 4 km SW de Huexca (18.7841 N, 98.888 W), 1368 m, 1 ♂ (MTO 930), 27/VII/2014, leg. M. Trujano-Ortega, U. Garcia; Huexca, Road 160, Cuautla-Izúcar, 2.5 km. NW de Tlayecac (18.7712 N, 98.8907 W), 1359 m, 3 ♂♂ (MTO 922, 924, 925), 27/VII/2014, leg. M. Trujano-Ortega, U. Garcia; Cañón de Lobos, 1 ♂ (CNIN-IBUNAM 27244), 15/II/1976, leg. J. Saldaña. **Veracruz (2)**: Naranjos, 8.3 km NE Rinconada (19.4018 N, 96.5272 W), 122 m, 1 ♂ (MTO 360), 3/VIII/2013, leg. M. Trujano-Ortega, A. Arellano, U. García; Tzonapa, Limonesintla (18.5293 N, 96.7999 W), 89 m, 1 ♂ (MTO 384), 4/IX/2013, leg. U. García. **Querétaro (1)**: Arroyo Seco, 3 km N Arroyo Seco (21.5655 N, 98.3310 W), 1097 m, 1 ♂ (MTO 1079), 15/IX/2014, leg. M. Trujano-Ortega, A. Arellano, O. Ávalos, U. García. **Hidalgo (1)**: Milpillas, Barranca de Meztitlan (20.5115 N, 98.6614 W), 1344 m, 1 ♂ (MTO 600), 31/V/2014, leg. U. García. **Oaxaca (1)**: Pluma Hidalgo, Rancho Hagia Sofia (15.8669 N, 96.3653 W), 406 m, 1 ♂ (MTO 253), 03/VII/2003, leg. A. Arellano. **Sinaloa (2)**: Puente Piaxtla (23.7486 N, 106.5951 W), 185 m, 1 ♂ (MTO 634), 8/VII/2014, leg. M. Trujano-Ortega, A. Arellano, U. García; Siqueros (23.3462 N, 106.244 W), 185 m, 1 ♂ (MTO 633), 8/VII/2014, leg. M. Trujano-Ortega, A. Arellano, U. García. **Jalisco (2):** Carr. 45 Aguascalientes-León, km 29, Lagos de Moreno (21.1376 N, 101.83 W), 1844 m, 1 ♂ (MTO 862), 20/VII/2014, leg. M. Trujano-Ortega, A. Arellano, U. García; Jamay (20.2944 N, 102.735 W), 1537 m, 1 ♂ (MTO 486), 31/III/2014, leg. M. Trujano-Ortega, A. Arellano, U. García.

***Emesis ares*. México: Sonora (1)**: 13 mi E of El Novillo, 1 ♂ (MZFC LEP 410225), 12/VIII/1985, leg. J. P. Brock.

***Emesis liodes*. México: Veracruz (1):** Teocelo, San Marcos, 1 ♂ (MZFC LEP 1914), 30/XI/1978, leg. J. Llorente.

**Emesis *mandana*. México: Jalisco (1)**: Ahutlan, Ahuacapan, 1 ♂ (ABD–123), 24/X/1989, leg. J. Llorente. **Tamaulipas (1):** San Marcos River, 1 ♂ (ABD–122), 29/XI/1986, leg. J. Kemner.

***Emesis saturata*. México: Morelos (1)**: Tepoztlán, Chichinautzin, km 85, (MZFC LEP 37476), 08/X/1995, leg. S. Valencia.

***Emesis tenedia.* México: San Luis Potosí (1)**: La Mera Ceiba, 1 ♂ (ABD–131), 08/I/1987, leg. J. Kemner.

***Neoapodemia chisosensis*. USA: Texas (2):** Crockett, Tx. 290, 1.6–1.1 road miles E. of Fort Lancaster, 2 ♂ (MGCL FL36, FL38), 2500ft 14/V/2007.

***Neoapodemia nais.* México: Durango (1**): El Madroño, 1 ♂ (MZFC LEP 2739) 27/VI/1987 leg. A. Luis, J. Llorente. **Chihuahua (1)**: Road La Providencia, 8/10 km W de Santa Bárbara (26.7724 N, 105.884 W), 2424 m, 1 ♂ (MTO 772), 2424 m, 16/VII/2014, leg. M. Trujano-Ortega, A. Arellano, U. García. **Sonora (1)**: Carr. Yécora-Cuauhtemoc, Km 290–291, Yécora (28.39581 N, 108.86584 W), 1583 m, 1 ♂ (MTO 706), 13/VII/2014 leg. M. Trujano-Ortega, A. Arellano, U. García. **USA: New Mexico (1):** Grant, Cherry Cr. Can., N. of Silver City, mile 11–12.5 on N. Mex. 15, 6950 ft, 1 ♂ (MGCL FL15), 03/VII/1989, leg. June Preston, Floyd Preston.
