# Supplementary material for: Two new genera of metalmark butterflies of North and Central America (Lepidoptera, Riodinidae)
Source: Zookeys. 2018 Jan 16;(729):61–85. doi: 10.3897/zookeys.729.20179 (PMC5799793; doi:10.3897/zookeys.729.20179)
Supplement: Supplementary material 2 — Collection and voucher data for Riodinids genetic samples used in this study [file zookeys-729-061-s002.docx]

**Supplement file SF 2. Collection and voucher data for Riodinids genetic samples used in this study.**

| No. | Voucher Number | Taxon | County | Locality | GenBank accesion number | | | Notes |
| --- | --- | --- | --- | --- | --- | --- | --- | --- |
|  |  |  |  |  | COI | Elongation | Wingless |  |
| 1 | RE01H224 | *Anteros allectus* | Costa Rica | Rio Taus: Cartago Province | KT286569 | KT286261 | KT286079 | GenBank |
| 2 | MTO202 | *Apodemia duryi* | México | Coahuila: Cuatro Ciénegas de Carranza |  |  |  |  |
| 3 | MTO510 | *Apodemia hepburni remota* | México | Baja California Sur: San Bartolo |  |  |  |  |
| 4 | MTO335 | *Apodemia h. hypoglauca* | México | Sinaloa: El Palmito |  |  |  |  |
| 5 | MTO336 | *Apodemia h. hypoglauca* | México | Sinaloa: El Palmito |  |  |  |  |
| 6 | MTO392 | *Apodemia h. hypoglauca* | México | Guerrero: Ixcateopan de Cuauhtémoc |  |  |  |  |
| 7 | MTO857 | *Apodemia h. hypoglauca* | México | Aguascalientes: Aguascalientes-León, km 116 |  |  |  |  |
| 8 | MTO393 | *Apodemia h. hypoglauca* | México | Guerrero: Ixcateopan de Cuauhtémoc |  |  |  |  |
| 9 | MTO344 | *Apodemia m. mexicanus* | México | Sonora: Carr. Mex. 45D. 46.2 km S de Hermosillo |  |  |  |  |
| 10 | MTO537 | *Apodemia m. mexicanus* | México | Baja California Sur: Loreto: 3 km SW de Loreto |  |  |  |  |
| 11 | MTO492 | *Apodemia m. mexicanus* | México | Baja California Sur: La Paz: 300 m S del CIBNOR |  |  |  |  |
| 12 | AS92Z472 | *Apodemia mormo* | USA | Lang Crossing: Nevada Count | KT286459 | KT286159 | KT285983 | GenBank |
| 13 | MTO394 | *Apodemia multiplaga* | México | Guerrero: Ixcateopan de Cuauhtémoc: Pipicantla |  |  |  |  |
| 14 | MTO261 | *Apodemia multiplaga* | México | Oaxaca: Pluma Hidalgo |  |  |  |  |
| 15 | MTO529 | *Apodemia murphy* | México | Baja California Sur: Los Cabos: Migriño |  |  |  |  |
| 16 | MTO706 | *Apodemia nais* | México | Sonora: Carr. Yécora-Cuauhtémoc, Km 290–291 |  |  |  |  |
| 17 | MTO772 | *Apodemia nais* | México | Chihuahua: 8–10 w of Santa Bárbara |  |  |  |  |
| 18 | MTO643 | *Apodemia palmeri arizona* | México | Sinaloa: El Fuerte: Sibajahui |  |  |  |  |
| 19 | MTO1209 | *Apodemia palmeri australis* | México | Querétaro: Cadereyta de Montes |  |  |  |  |
| 20 | MTO214 | *Apodemia palmeri australis* | México | Coahuila: Cuatro Ciénegas de Carranza: Tío Cándido |  |  |  |  |
| 21 | MTO235 | *Apodemia palmeri australis* | México | Coahuila: Cuatro Ciénegas de Carranza: Pozas Azules |  |  |  |  |
| 22 | MTO303 | *Apodemia palmeri australis* | México | Durango: Nombre de Dios |  |  |  |  |
| 23 | MTOH84 | *Apodemia phyciodoides* | México | Sonora: Yécora-Cuauhtémoc, Km 300–302, Los Pilares |  |  |  |  |
| 24 | ABDRIOD69 | *Apodemia walkeri* | México | Morelos |  |  |  |  |
| 25 | MTO1079 | *Apodemia walkeri* | México | Querétaro: Arroyo Seco: 3 km N de Arroyo Seco |  |  |  |  |
| 26 | MTO486 | *Apodemia walkeri* | México | Jalisco: Jamay: Jamay |  |  |  |  |
| 27 | MTO862 | *Apodemia walkeri* | México | Jalisco: Carr. 45 Aguascalientes-Leon, km 29 |  |  |  |  |
| 28 | RE01H221 | *Baeotis nesaea* | Costa Rica | Rio Taus: Cartago Province | KT286568 | KT286260 | KT286078 | GenBank |
| 29 | JH03R041 | *Calydna sturnula* | Ecuador | Cerro Lumbaqui Norte: Su | KT286504 | KT286199 | KT286026 | GenBank |
| 30 | PDV94A005 | *Detritivora gynaea* | Panamá | Barro Colorado Island | KT286532 | KT286225 | KT286052 | GenBank |
| 31 | RE06E140 | *Dodona elvira* | Indonesia | NE Kalimantan | KT286595 | KT286285 | KT286103 | GenBank |
| 32 | PDV94B017 | *Emesis lucinda* | Ecuador | Sucumbios: Garza Cocha: Anangu, 175 km of Coca | KT286551 | KT286244 | KT286067 | GenBank |
| 33 | PDV94T022 | *Emesis mandana* | México | Sucumbios: Garza Cocha: Anangu, 175 km of Coca | KT286562 | KT286254 | KT286073 | GenBank |
| 34 | MTO385 | *Emesis* sp. | México | Veracruz: Tzonapa: Limonesintla |  |  |  |  |
| 35 | MTO357 | *Emesis* sp. | México | Veracruz: : Naranjos, 8.3 km al Noreste de Rinconada |  |  |  |  |
| 36 | MTO478 | *Emesis* sp. | México | Nayarit: Tepic: Carr. Mex. 15, 12.2 km NW de Tepic |  |  |  |  |
| 37 | PDV94D002 | *Esthemopsis jesse* | Ecuador | Sucumbios: Garza Cocha: Anangu, 175 km of Coca | KT286556 | KT286249 | KT286070 | GenBank |
| 38 | RE01H159 | *Eurybia unxia* | Costa Rica | Rio Grande Orosi: Cartago Province | KT286565 | KT286257 | KT286076 | GenBank |
| 39 | MFB00T814 | *Euselasia hieronymi* | Costa Rica | Colon | KT286579 | KT286271 | KT286088 | GenBank |
| 40 | RE01H229 | *Hades noctula* | Costa Rica | Rio Taus: Cartago Province | KT286570 | KT286262 | KT286080 | GenBank |
| 41 | PDV94A021 | *Helicopis cupido* | Ecuador | Sucumbios: Garza Cocha: Anangu, 175 km of Coca | KT286536 | KT286229 | KT286055 | GenBank |
| 42 | JH03R027 | *Ithomiola tanos* | Ecuador | Near Baeza, NA | KT286492 | KT286192 | KT286014 | GenBank |
| 43 | AS92Z211 | *Lycaena arota* | USA | Topaz Lake: Mono County: California | KT286458 | KT286158 | KT285982 | GenBank |
| 44 | MAT98V738 | *Melanis pixe* | Panamá | Gamboa: 183 Smithsonian Apartment building | KT286507 | KT286202 | KT286029 | GenBank |
| 45 | JH03R019 | *Mesene nola* | Ecuador | Misahualli, WA | KT286484 | KT286184 | KT286006 | GenBank |
| 46 | PDV94A033 | *Nymphidium* sp. | Ecuador | Sucumbios: Garza Cocha: Anangu, 175 km of Coca | KT286541 | KT286234 | KT286059 | GenBank |
| 47 | MWT93A074 | *Paralaxita damajanti* | Malaysia | Kepong | KT286520 | KT286213 | KT286040 | GenBank |
| 48 | NK00P826 | *Polycaena temerlana* | Kazakhstan | Bolshaya: Almatinka | KT286526 | KT286219 | KT286046 | GenBank |
| 49 | 05SRNPP30061 | *Sarota chrysus* | Costa Rica | Guanacaste: Sector Pitilla: Pasmompa | KT286583 | KT286274 | KT286092 | GenBank |
| 50 | PDV94B030 | *Sarota gyas* | Ecuador | Sucumbios: Garza Cocha: Anangu, 175 km of Coca | KT286555 | KT286248 | KT286069 | GenBank |
| 51 | JH03R026 | *Siseme aristoteles* | Ecuador | Near Baeza | KT286491 | KT286191 | KT286013 | GenBank |
| 52 | PDV94B003 | *Stalachtis calliope* | Ecuador | Sucumbios: Garza Cocha: Anangu, 175 km of Coca | KT286546 | KT286239 | KT286063 | GenBank |
| 53 | MWT93B071 | *Stiboges nymphidia* | Malaysia | Genting Tea Estate | KT286522 | KT286215 | KT286042 | GenBank |
| 54 | GL02N259 | *Styx infernalis* | Perú | Cuzco: Valle de Cosnipata, ca. 3 km W. San Pedro | KT286466 | KT286166 | KT285989 | GenBank |
| 55 | PDV94A024 | *Synargis abaris* | Ecuador | Sucumbios: Garza Cocha: Anangu, 175 km of Coca | KT286539 | KT286232 | KT286057 | GenBank |
| 56 | MWT93A019 | *Zemeros flegyas* | Malaysia | Kepong | KT286514 | KT286207 | KT286034 | GenBank |
