# Supplementary figures and images for: Two new genera of metalmark butterflies of North and Central America (Lepidoptera, Riodinidae)
Source: Zookeys. 2018 Jan 16;(729):61–85. doi: 10.3897/zookeys.729.20179 (PMC5799793; doi:10.3897/zookeys.729.20179)

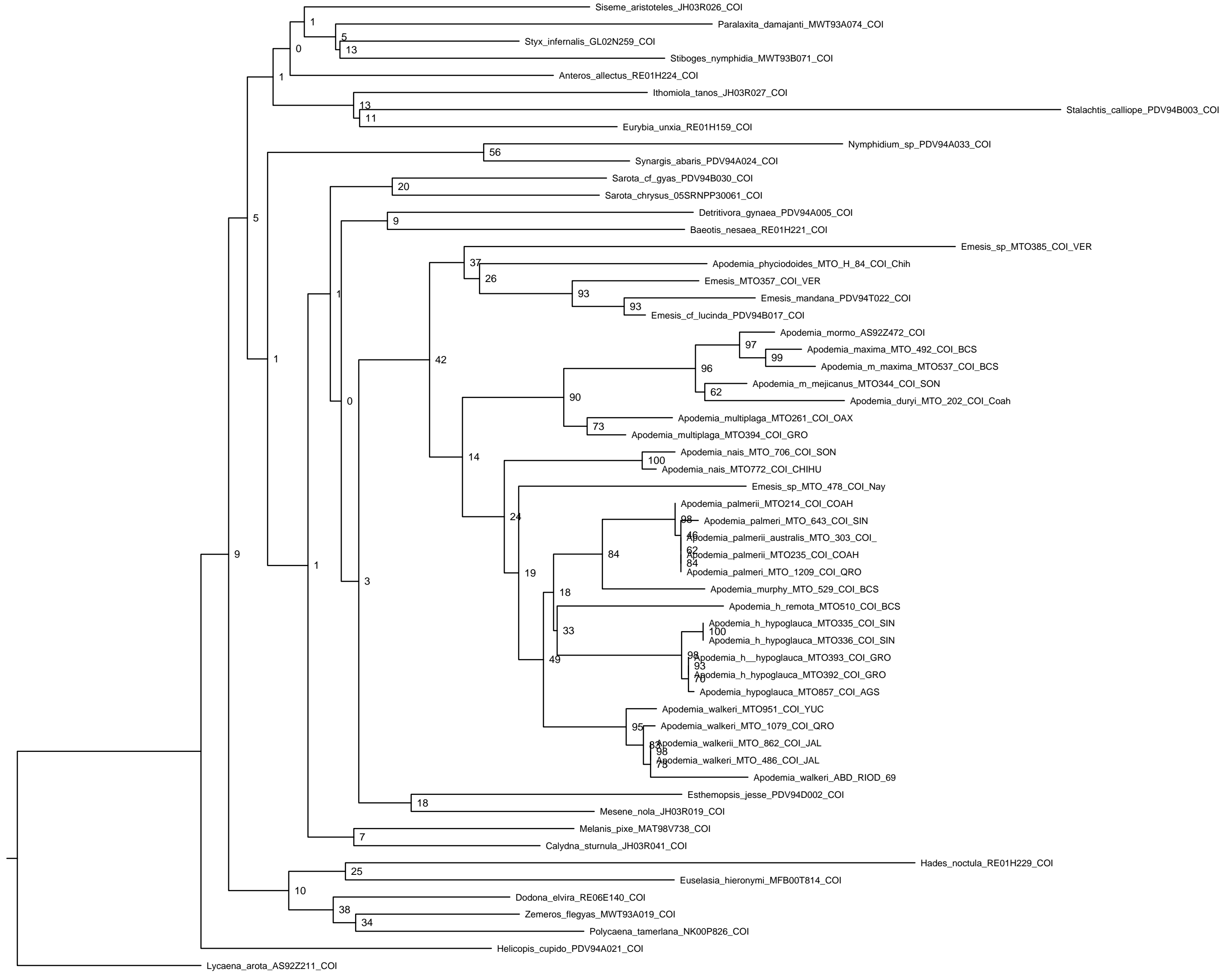

Supplement: Supplementary material 3 — Maximum likelihood tree of the relationships among Apodemia and selected species in the Riodinidae inferred with Cytochrome Oxidase I (COI). Numbers near branch nodes are bootstrap branch support [file zookeys-729-061-s003.pdf]

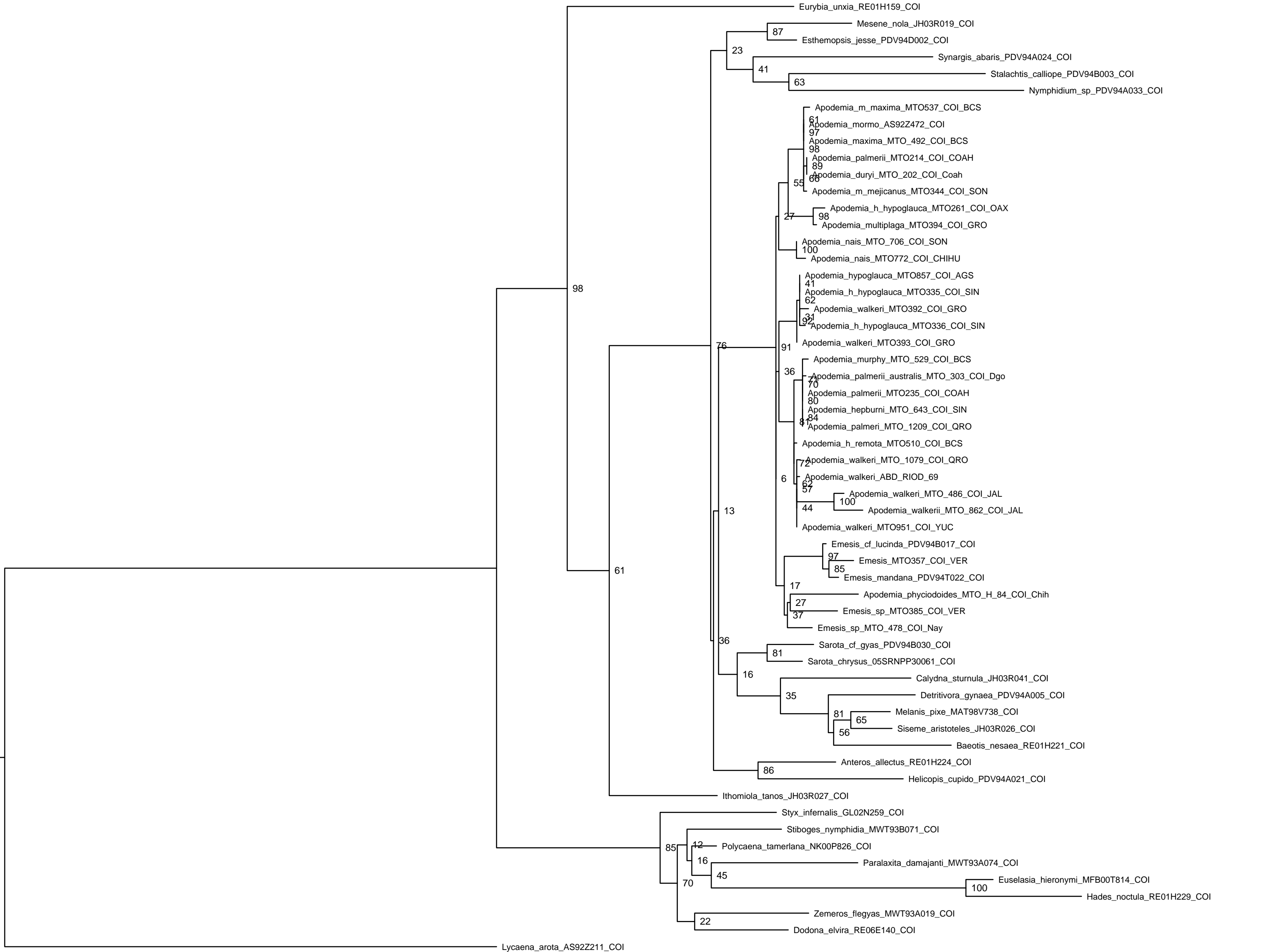

0.1

Supplement: Supplementary material 4 — Maximum likelihood tree of the relationships among Apodemia and selected species in the Riodinidae inferred with Wingless (wg). Numbers near branch nodes are bootstrap branch support [file zookeys-729-061-s004.pdf]

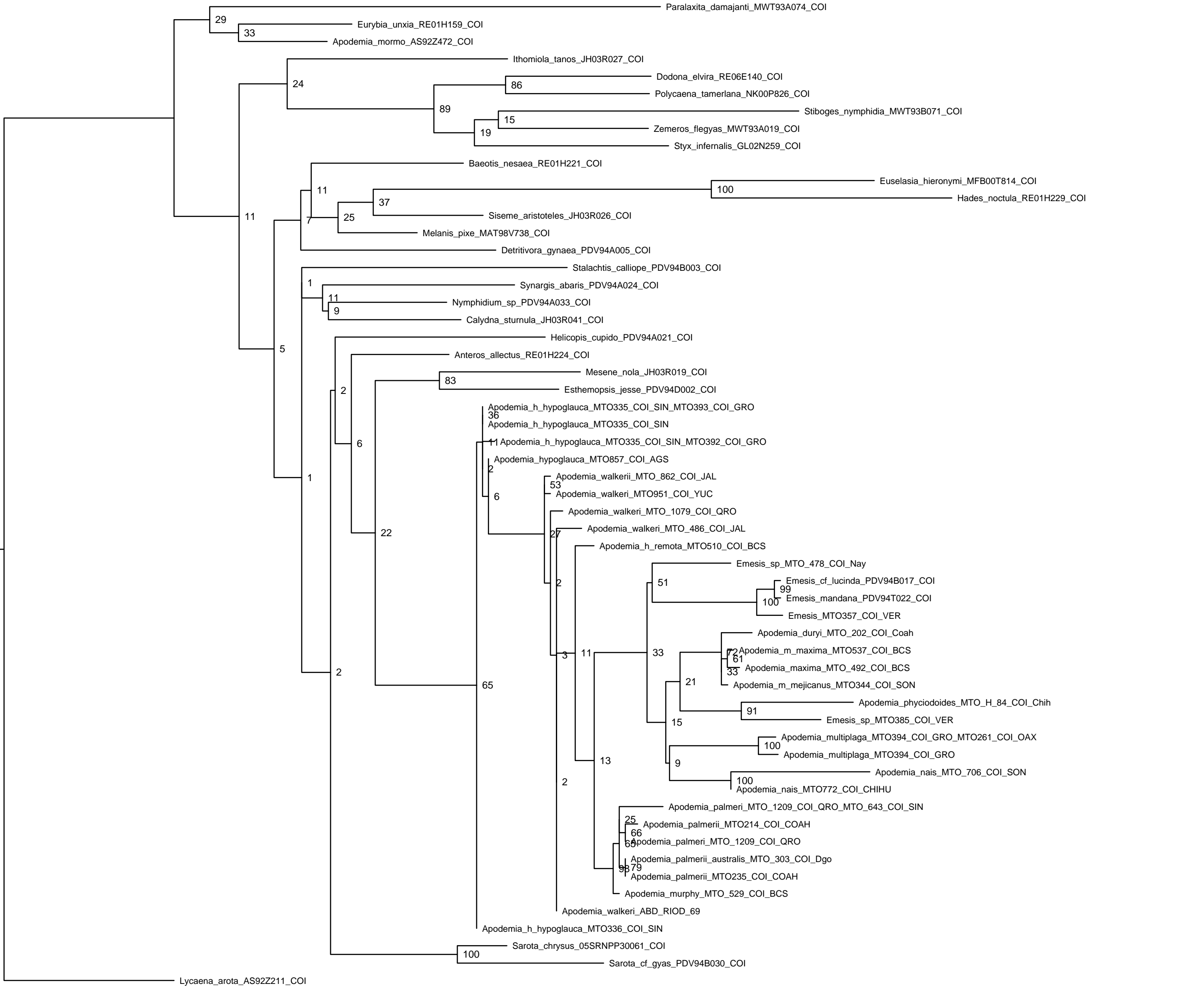

0.04

Supplement: Supplementary material 5 — Maximum likelihood tree of the relationships among Apodemia and selected species in the Riodinidae inferred with gene Elongation factor 1 α (EF-1a). Numbers near branch nodes are bootstrap branch support [file zookeys-729-061-s005.pdf]
