# Supplementary material for: Two new genera of metalmark butterflies of North and Central America (Lepidoptera, Riodinidae)
Source: Zookeys. 2018 Jan 16;(729):61–85. doi: 10.3897/zookeys.729.20179 (PMC5799793; doi:10.3897/zookeys.729.20179)
Supplement: Supplementary material 6 — Data of the photographs shown in the Figure 6 [file zookeys-729-061-s006.docx]

**Supplementary file SF 6: Data of the photographs in the Figure 6.**

**A) *Neoapodemia chisosensis***. HOLOTYPE ♂ USA: TEXAS: Chisos Mtns., 03/VIII/1962 [AMNH]. Nick V. Grishin. **B) *Neoapodemia nais***. ♂ MEXICO: CHIHUAHUA: Madera, June 1956 [MGCL]. Kim Davis, Mike Stangeland and Andrew Warren. **C) *Plesioarida h. hepburni*.** ♂ MEXICO: SONORA: Trinidad, 10-X-88 [MGCL] Kim Davis, Mike Stangeland and Andrew Warren. **D) *Plesiorida murphy***. HOLOTYPE ♂ MEXICO: BAJA CALIFORNIA SUR: Arroyo San Bartolo, 28/VIII/82 [SDNHM]. Kim Davis, Mike Stangeland and Andrew Warren. **E) *Plesioarida h. hypoglauca***. ♂ MEXICO: SONORA: Rute 16, 3 mi W of Trinidad, 08/VIII/86 [MGCL]. Kim Davis, Mike Stangeland and Andrew Warren. **F) *Plesioarida p. palmerii***. ♂ Afton County Park, San Bernardino Co., CA, USA 23/VIII/75. Jim P. Brock. **G) *Plesioarida walkeri*.** ♂ St. Hwy. 18, 10–30 Km. West of Cosala, SIN, MX 16/I/79. Jim. P. Brock. **H) *Apodemia m. mormo***. ♂ USA: CALIFORNIA: Nevada Co., 2 mi N of Hwy 20 on Bowman Lake Road, 24/VIII/81 [MGCL]. Kim Davis, Mike Stangeland and Andrew Warren. **I) *Apodemia duryi*.** ♂ Brewster Co., TX., USA 30/V/99. Jim. P. Brock. **J) *Apodemia m. mejicanus.*** ♂ vicinity of Nacapuli Canyon, San Carlos, SON, MX 25–26/III/03. Jim. P. Brock. **K) *Apodemia multiplaga***. ♂ playa, 1 Km East of Bahia San Carlos, SON, MX 21/III/98. Jim P. Brock. **L) *Apodemia v. virgulti***. (NEOTYPE of *Nemeobius virgulti*) USA: CALIFORNIA: Los Angeles Co., La Tuna Canyon, 27/IX/1951 [CAS]. Qian Cong and Nick V. Grishin.
